# Sustained mood improvement with laughing gas exposure (SMILE): a randomised, placebo-controlled pilot trial of nitrous oxide for treatment-resistant depression: commentary, Kalmar et al

**DOI:** 10.1192/bjo.2026.12040

**Published:** 2026-07-03

**Authors:** Alain F. Kalmar, Pascal Sienaert, Filip Bouckaert, Steffen Rex

**Affiliations:** Electronics and Information Systems, Ghent University Faculty of Engineering and Architecture, Belgium; Anaesthesia, Intensive Care and Pain Medicine, AZ Maria Middelares, Ghent, Belgium; Department of Mood Disorders, University Psychiatric Centre KU Leuven, Belgium; Old Age Psychiatry, University Psychiatric Centre KU Leuven, Belgium; Anaesthesiology, University Hospitals Leuven, Belgium

**Keywords:** Nitrous oxide, treatment-resistant depression, N_2_O, climate change, sustainability

## Abstract

Nitrous oxide is being investigated as a treatment for therapy-resistant depression, yet its environmental implications as a potent greenhouse gas are largely unaddressed. A single 1 h treatment generates ∼150 kg CO_2_-equivalents, rising to ∼7.8 t per patient-year, highlighting the need to incorporate environmental externalities into evaluation.

**Figure:**
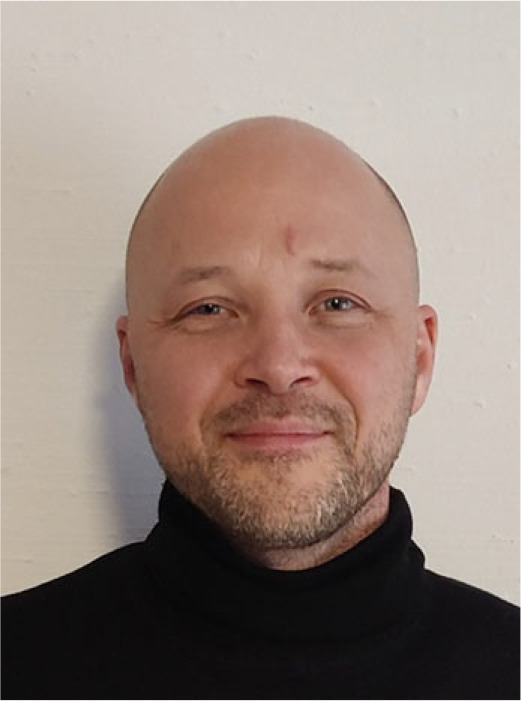

In a recent article in *BJPsych Open*, Ladha et al reported a randomised, placebo-controlled pilot study suggesting that repeated nitrous oxide (N_2_O) inhalation may offer symptomatic relief in treatment-resistant depression (TRD).^1^ The study demonstrates feasibility and acceptable tolerability of 4 weekly 1 h sessions of 50% N_2_O, supporting the rationale for a definitive multicentre trial. However, an important dimension relevant to psychiatric practice and policy – namely the environmental implications of scaling such treatment – remains unaddressed.

N_2_O is not only a long-standing anaesthetic gas but also a potent and persistent greenhouse gas. With an atmospheric lifetime exceeding a century and a 100-year global-warming potential (GWP_100_) of 273, N_2_O currently accounts for approximately 6% of total anthropogenic radiative forcing and is also the single most significant ozone-depleting emission of the 21st century.^2^

Although medical N_2_O use is small relative to agricultural and natural sources, this comparison risks obscuring the disproportionate footprint of individual therapeutic decisions within healthcare. On a global scale, almost any individual activity appears negligible; nevertheless, from the perspective of personal accountability, a single therapeutic choice can carry a disproportionately large and ethically difficult-to-justify footprint, particularly when the clinical benefits are not of comparable magnitude.

Using the parameters of the SMILE protocol – 50% N_2_O for 1 h at an assumed 10 L/min gas flow – one session entails delivery of roughly 549 g of N_2_O. This figure follows directly from the universal gas law (1 mol = 24.06 L at 20 °C, 1 atm): 5 L/min corresponds to 0.208 mol/min = 9.15 g/min, or 549 g/h. Multiplied by the GWP_100_ of 273, each treatment equals ∼150 kg CO_2_-equivalents (CO_2_e). Even a short maintenance course of 4 weekly sessions would thus emit about 600 kg CO_2_e per patient. If extended as a long-term therapy – weekly sessions over 1 year – annual emissions would reach ≈7.8 t CO_2_e per patient, substantially exceeding the average per capita greenhouse gas footprint of a European citizen (5.86 t CO_2_e/year).

Although such calculations are approximate, they underscore the magnitude of the problem. Unlike other sources of N_2_O, these emissions would occur directly from healthcare facilities, without feasible recovery or abatement. Anaesthetic scavenging systems are designed primarily for occupational safety and do not destroy N_2_O; catalytic destruction units exist but are expensive, rarely installed and are not optimised for low-pressure, intermittent psychiatric use.^3^ In other words, nearly all gas administered in this context will reach the atmosphere unchanged.

For perspective, manufacturing a full 50 mg therapeutic dose of esketamine entails only about 7 g CO_2_e, a figure several thousand times lower than that of one N_2_O session.^4^ Thus, while the authors appropriately discuss feasibility and tolerability, the feasibility of scaling such a therapy at population level must also be judged against its planetary health cost.

It may be argued that mental health crises justify environmental compromise. However, the ethical calculus should recognise that N_2_O’s long atmospheric lifetime makes its consequences intergenerational: each kilogram emitted today will continue to warm and deplete ozone well into the next century. This cannot easily be offset or reversed. If psychiatric indications for N_2_O were to proliferate – potentially involving a substantial proportion of patients with depressive disorders receiving recurrent high-flow exposures – the cumulative footprint could rival that of all anaesthetic N_2_O.

Notably, the original trial required approximately 2 years to recruit 40 patients, underscoring the early stage of clinical implementation. Although electroconvulsive therapy (ECT) represents the most established intervention for treatment-resistant depression, its utilisation is constrained by the need for general anaesthesia and specialised infrastructure; if N_2_O were to demonstrate clinical efficacy, its simpler administration could allow for broader uptake although the eventual scale of use remains uncertain.

For example, in Belgium the total national medical consumption of N_2_O dropped from 300 t in 2015 to 94 t in 2024, a figure that is rapidly declining.^5^ A weekly treatment session using the SMILE protocol consumes approximately 549 g of N_2_O, corresponding to about 28 kg per patient per year. If an estimated 3600 patients – equivalent to roughly 1 patient per 3200 citizens – were to receive maintenance therapy, the resulting annual emissions would already exceed the 2024 national medical total of 94 t. These illustrative calculations demonstrate how rapidly psychiatric indications alone could reshape national medical N_2_O emissions if adopted at scale. For additional context, 1018 patients underwent ECT in Belgium in 2023 (approximately 1 per 10 000 citizens). Given the substantially lower procedural, logistical and regulatory barriers associated with inhaled N_2_O, broader uptake for depressive disorders could plausibly exceed this benchmark, with corresponding implications for cumulative emissions.

At the same time, the therapeutic landscape for treatment-resistant depression is rapidly evolving, with multiple emerging modalities under investigation; consequently, the eventual uptake of any single intervention remains uncertain. Should such use be implemented at scale, it could rapidly amplify national medical N_2_O emissions beyond those illustrated above.

The interface between climate change and mental health has received increasing attention in recent years.^6–8^ Notably, The Royal College of Psychiatrists’ Sustainability Committee has generated a summary of ten ways to reduce one’s footprint in the professional setting, acknowledging that continuing clinical practice that ignores environmental impact will contribute to an international mental health crisis.^9^ Within this context, the environmental externalities of emerging psychiatric therapies warrant explicit consideration. In addition, exposure-related risks, including N_2_O use disorder, have been described in medical settings.^10^

Moreover, the environmental externalities are entirely absent from cost-effectiveness analyses. Within a ‘triple bottom line’ framework – balancing patient outcomes, environmental impact and economic cost – current analyses suggest that direct procedural costs are broadly comparable between strategies; however, these assessments omit the societal cost of carbon: applying recent meta-analytic estimates of ∼US$700–900 per ton CO_2_ translates the emissions of a single N_2_O treatment (∼150 kg CO_2_e) into an additional societal cost of ≈US$120 per session, rising to over US$6000 per patient-year – costs that are externalised to future generations and disproportionately borne by vulnerable populations and natural ecosystems.^11,12^

In addition, occupational exposure to N_2_O remains a recognised concern, with recent evidence supporting a health-based exposure limit of approximately 20 mg/m^3^ (8 h time-weighted average), implying the need for dedicated ventilation and scavenging systems; tightening exposure standards may therefore render large-scale implementation increasingly complex and costly.

In anaesthesia, health systems are already phasing out N_2_O owing to its climatic impact. Extending medical N_2_O use to chronic psychiatric therapy would run counter to this broader decarbonisation trajectory. There is, therefore, a compelling case that any further clinical research on N_2_O for depression should quantify not only symptom change but also life cycle greenhouse gas emissions, expressed in CO_2_e per treatment and per patient-year. Accordingly, environmental impacts should be explicitly incorporated into research protocols, with prospective quantification of greenhouse gas emissions and structured consideration of these data when weighing the benefits and harms of N_2_O as a therapeutic option. However, how such environmental impacts should be formally weighed against clinical benefits remains an open question, with no widely adopted frameworks currently available to guide such decisions.

Future investigators might also explore low-flow or closed-circuit delivery systems analogous to those used in modern anaesthesia, which can reduce N_2_O consumption by more than 90%. However, even under ideal conditions, the residual climate impact would remain substantial compared with non-gaseous alternatives. Unless a practical and scalable destruction or capture technology is implemented – potentially justified only in the context of substantial and clearly demonstrated clinical benefit, and a challenge even in well-equipped operating rooms – routine psychiatric use of N_2_O risks creating a new category of high-emission therapy within medicine.

This Commentary does not question the clinical need for innovation in TRD, nor the promising neurobiological mechanisms of N_2_O.^13^ Rather, it highlights the importance of integrating environmental accountability into the early evaluation of interventions whose primary agent is itself a greenhouse gas. Explicit consideration of such externalities is consistent with the profession’s broader commitment to minimising harm – both to patients, particularly in carefully selected cases, and, increasingly, to public and planetary health.
